# Predictors of Interest in an Alcohol Reduction Clinical Trial of Naltrexone among Undergraduates

**DOI:** 10.4172/2155-6105.1000151

**Published:** 2013-05-24

**Authors:** Robert F Leeman, William R Corbin, Lisa M Fucito, John W Urwin, Stephanie S O’Malley

**Affiliations:** 1 School of Medicine, Yale University, USA; 2 Arizona State University, USA

**Keywords:** Alcohol-related consequences, College drinking, Heavy drinking, Impaired control over alcohol use, Pharmacotherapy, Smoking, Web survey

## Abstract

**Background:**

We tested predictors of interest in a clinical trial of naltrexone plus counseling for heavy drinking reduction in young adults using a web survey. Respondents could indicate interest in the clinical trial at the conclusion of the survey.

**Methods:**

A random sample of university students completed the survey (*N* = 584, 60% female). Data were collected in October-November 2010.

**Results:**

Among past-year drinkers (*n* = 411), 22.6% (*n* =93) indicated interest. Equivalent levels of interest were found among past-year heavy drinkers. Non-white race and current cigarette smoking predicted interest. Alcohol-related negative consequences score was a trend-level predictor in the full regression model, but a significant predictor in a reduced model.

**Conclusions:**

Non-white students, smokers and those with a high number of negative consequences may be more amenable to drinking reduction via medication and counseling. These findings could facilitate efforts of researchers, administrators, counselors and other professionals to tailor drinking reduction messages and facilitate treatment engagement by undergraduates.

## Introduction

Heavy drinking among undergraduates is a major public health concern. According to the National Epidemiologic Survey on Alcohol and Related Conditions (NESARC), 31.3% of undergraduate, past-year drinkers reported two or more heavy episodic drinking occasions per month and 16.7% reported two or more occasions per week [1]. Heavy episodic drinking has been linked to negative consequences, including fatal traffic accidents [2]. Alcohol use disorders (AUD; i.e., abuse or dependence) are also common among undergraduates, with a national survey finding that 24% of men and 13% of women met criteria for a current AUD [3]. Both rates are considerably higher than national averages.

Although heavy drinking and AUD rates are high, evidence suggests young people are reticent to engage in mental health treatment in general and for addictive behaviors, in particular. Based on analyses of NESARC data, Blanco et al. [4] found that about 45% of college students met current criteria for at least one psychiatric disorder, however less than 20% of those individuals had received any mental health treatment in the past year. In a survey at 26 American colleges, 32% were classified as having a mental health problem, but only 36% of these individuals had received any treatment in the past year [5]. Pertaining to alcohol problems specifically, Wells et al. [6] found that, among 25 year olds in New Zealand, only 7% who reported an alcohol-related problem sought alcohol treatment in the prior four years. At a state university in the United States, only 18% of current heavy drinkers reported having ever utilized any of 14 drinking reduction resources [7]. Among current drinkers at another state university in the U. S., only 14.6% reported interest in cutting down their alcohol use and only 5.4% indicated interest in stopping [8]. There are many reasons for undergraduates’ reticence to change. These include a lack of interpersonal reasons for quitting (i.e., a lack of social control from parents and other adults) [9] and an attitude that their drinking behavior is not problematic [6,10,11].

The nature of the relationship between alcohol problem severity and treatment interest is unclear. Some studies have found that alcohol problem severity predicts treatment interest and help-seeking. Wells et al. [6] found that treatment seeking increased to 24% among those meeting alcohol dependence criteria and Cellucci et al. [10] reported a significant, positive correlation between AUDIT scores and help-seeking interest. Providing further evidence that greater problem severity is associated with greater interest in treatment, Epler et al. [8] found that the number of alcohol dependence symptoms endorsed significantly predicted interest in oral medication as needed for drinking situations among undergraduates. In the same survey, negative consequences predicted interest in daily oral medication use. At the same time, other studies have found interest in brief interventions to be weaker among heavier drinking undergraduates [12,13]. Buscemi et al. [7] found equivocal results in that there were positive relationships between negative consequences and past help seeking, but negative relationships with hypothetical future help seeking. Further, Neighbors et al. [14] found an inverted U-shaped relationship between drinking severity and interest in behavior change. The lightest and heaviest drinkers reported the least interest and the heaviest drinkers were also unlikely to actually participate. They attributed these findings to a lack of perceived need among light drinkers and possible defensiveness among heavier drinkers.

Young adults may prefer interventions to reduce consumption and associated harms rather than to promote outright abstinence [8], and evidence supports the efficacy of individual alcohol reduction counseling in undergraduates. However, effect sizes are modest, and these interventions may not be as efficacious for the heaviest drinkers [15]. The addition of pharmacotherapy may increase effect sizes. With psychotropic medication use for other disorders becoming more common among undergraduates, health care professionals may be more likely to consider medication for young heavy drinkers. Indeed, in a sample of 26 colleges, 13.7% reported past year psychotropic medication use, a prevalence just under counseling/psychotherapy (14.8%) [5]. It is valuable to know the extent to which students are open to pharmacotherapy for drinking reduction, along with characteristics of those who are more and less interested in medication for this indication.

The opiate antagonist naltrexone may represent the best option for pharmacotherapy for alcohol use reduction [16]. Findings support naltrexone’s efficacy in alcohol treatment [17] and it might be particularly well suited to young adults, who may be unwilling to abstain [8]. Naltrexone is safe to take while consuming alcohol and has been shown to be efficacious for reducing drinking in heavy drinkers not interested in stopping [18]. In contrast, disulfiram’s mechanism of action precludes its use to promote moderate drinking [19] and evidence suggests that treatment with acamprosate is most efficacious in promoting alcohol abstinence [20]. Long-acting, injectable naltrexone would seem to be a viable option given that it was designed to mitigate issues of questionable motivation and medication adherence [21], but undergraduates in a recent survey expressed less interest in injectable medications for drinking reduction than in oral medications [8]. Although there is limited research in young adults, a small, open-label pilot study provided evidence for feasibility and preliminary efficacy of naltrexone, plus individual counseling [22]. In addition, the National Institute on Alcohol Abuse and Alcoholism (NIAAA) has recognized naltrexone’s value for young adults. Former NIAAA director of the division of treatment and recovery research, Dr. Mark Willenbring, stated “One of my goals is to make naltrexone the hottest drug on campus” [23] (p 1).

Researchers have recognized that “finding methods to increase treatment utilization and retention among emerging adults and adolescents is a research priority” [9] (p 400). One method to increase resource utilization among college students is to assess which characteristics are associated with openness to treatment. This information could be used by researchers, counselors and health professionals who work with this population to target students who may be more open to changing their behavior. At the same time, different and perhaps more aggressive approaches may be needed to target students without these characteristics.

Given the established efficacy of individual counseling and naltrexone, it would be valuable to determine which characteristics are associated with interest in a clinical trial testing this combination of approaches. Given modest prescription rates for naltrexone [24], it is unlikely that many young adults are knowledgeable about the indications for naltrexone. Therefore, openness to treatment with naltrexone and individual counseling could serve as a proxy for willingness to engage in a more intensive intervention. A survey to predict interest in a clinical trial of counseling and naltrexone is unique in that little is known about undergraduates’ willingness to take medication for alcohol use reduction, with only one recent survey addressing this topic [8]. Further, most surveys concern prior involvement [6,7,11] and/or hypothetical future interest in treatment [7,8,10]. In the present study, respondents declaring interest knew that they would be contacted by study staff. Thus, respondents were taking a form of action by indicating interest.

As in previous studies, alcohol use and negative consequences were considered as possible predictors of interest. Several additional variables that have been linked to greater problem drinking severity were also included as possible predictors of interest. These risk factors include male sex (e.g., [25]); white race [26]; residing in a dormitory or other dwelling without parents [27]; underclass academic status ([25], though see [28] for evidence of more severe drinking among older students); drug use [29] and emotional/psychiatric difficulties [30]. Given the paucity of reports concerning interest in treatment and in clinical trial participation in college students, no definitive predictions were made regarding the pattern of results.

Finally, smoking status and perceived peer alcohol use were included as potentially important predictors of interest. Cigarette smoking has been related to an increased likelihood of problem drinking [31] and poorer alcohol treatment outcomes [32]. Drinking behavior change may be particularly challenging for smokers and as a result, they may perceive greater need for assistance in reducing their drinking and show more interest in an alcohol reduction clinical trial. In contrast, undergraduates with perceptions of heavier drinking among their peers may view their own drinking as more normative and may perceive less need to change their drinking behavior [33].

## Methods

### Participants and procedures

A web-based survey was conducted at a small, private university in the Northeastern United States. A random sample of 1,822 students was notified about an upcoming survey via an email from the university’s counseling office. The sex and academic class (i.e., freshman, sophomore, junior and senior) composition of the invited sample was commensurate with the student body at large. One week later, an email including the survey link was distributed. A coupon for a free coffee was included, regardless of participation. Two reminder emails were sent in the 18 days the survey was open. Respondents were also enrolled in drawings for $25 (1 in 20 odds) and $500 (1 winner) gift cards.

Respondents had to be at least 18 years old. During informed consent, respondents were notified that they could have their responses considered for preliminary eligibility for a clinical trial testing “individual counseling and an FDA-approved medication called naltrexone.” This statement was reiterated at the end of the survey, followed by the statement “Participants in this study are compensated up to $470 in the course of the study.” The web address for the clinical trial was provided so students could obtain more information. This survey and clinical trial were approved by the Institutional Review Board (IRB) of the Yale University School of Medicine and the survey was approved by the IRB of the university where the survey was conducted.

### Measures

Efforts were made to keep the survey brief to enhance response rates. A number of items concerned recent substance use. Past-year alcohol use was measured with five items from question sets recommended by NIAAA [34]. Two of these items were selected for analysis in the present report: 1) an item concerning how often they consumed five drinks or more for males and four drinks or more for females within a two hour period in the past 12 months (i.e., binge drinking) and 2) an item concerning the number of alcoholic drinks they had on a typical day when they drank alcohol (i.e., drinks per drinking day) in the past 12 months. A categorical range of responses was provided for each item (e.g., 7 to 8 drinks). Scores were obtained by taking the average of each range (e.g., 7.5). Binge drinking scores were converted to weekly estimates. Similar averaging procedures have been utilized with these types of items by other investigators [35] and have been deemed to yield reliable estimates [36]. Based on subscales of the Young Adult Alcohol Consequences Questionnaire (YAACQ) [37], we created a very brief measure of alcohol-related negative consequences. An item was written to exemplify each of the 8 subscales of the YAACQ. To this we added an item pertaining to negative sexual consequences (Table 1). Respondents indicated whether or not they experienced each consequence in the past three months. A sum score was derived (*alpha* = 0.78). Participants were asked to report whether or not they smoked cigarettes at least once per week. Participants also reported whether or not they had used marijuana or other illegal drugs at all in the past year and also whether they had used prescription medication not as prescribed in the past year.

Respondents also completed items concerning their perception of the typical drinking quantity per occasion of three classes of peers: 1) their friends; 2) other, same aged male students and 3) other, same-aged female students. Options for each item ranged from “0” to “20 or more” drinks. An average of these three items was taken (*alpha* = 0.90). The survey also included demographic items (e.g., race/ethnicity, sex), an item pertaining to current living situation (i.e., dormitory, residence with friends/roommates, or residence with parents) and a binary, yes/ no item concerning current treatment for “emotional or psychiatric difficulties.”

The first stage of the analysis plan involved examination of demographic characteristics of the surveyed sample compared to the full student body to assess representativeness. We then examined descriptives for key variables in the entire sample. Only those reporting any past-year alcohol consumption were included in subsequent analyses since interest in clinical trial participation was relevant only to this subset of students. Next, we examined the distributions of all continuous variables and conducted preliminary analyses including an examination of correlation among the study variables. The primary analyses were conducted using binary logistic regression to predict interest in clinical trial participation. Given that respondents were informed at the outset about the opportunity for their responses to be evaluated for preliminary eligibility for the clinical trial, we treated omissions on the “interest” item as “no” responses. All relevant predictor variables were entered into an initial regression model and then a second model was tested, involving only statistically significant and near-significant predictors. Field [38] advocated for this type of two-tiered approach to regression analysis. Given prior empirical results suggesting an inverted, U-shaped curve in relationships between alcohol consumption and clinical trial interest, in which weaker interest was found among the heaviest and lightest drinkers [14], both linear and quadratic terms were considered for the alcohol consumption variables included in the regression models.

## Results

### Representativeness of survey participants

Informed consent was given by 584 students for a response rate of 32.1%. While this rate was low, several recent, web-based surveys concerning substance use have reported comparable response rates in undergraduates: Lange et al. [39] 25.3%, 18.2% and 20.6% in separate surveys; McCabe et al. [40] response rates at individual schools ranged from 14.8%−46.1% with 5 of 8 schools in the 30% range; O’Brien et al. [41] targeted a 33% response rate; Palmer et al. [42] 29.4%; Shillington et al. [43] 32%. These surveys all offered compensation that was comparable to the present study.

Sample descriptives can be found in Table 1. The sample was mainly White and had a majority of women, who were somewhat over-represented given that they represented 49.9% of the total student body. The racial/ethnic breakdown was comparable to the student body except that Hispanic participants were over-represented as only 6% of the total student body identified as such. The breakdown of the sample by academic class was comparable to the student body.

### Other characteristics of survey participants

Past year alcohol use was reported by 70.4% (*n* = 411) of students. In the full sample, men reported a mean of 4.19 drinks per drinking day (*SD* = 4.54), with women reporting a mean of 2.47 (*SD* = 2.45). Men reported a mean of 0.68 binge episodes per week (*SD* = 1.22), with women reporting 0.31 (*SD* = 0.69) binge episodes. Among past-year drinkers, mean drinks per drinking day was 5.85 (*SD* = 4.37) for men and 3.43 (*SD* = 2.25) for women. Frequency of binge drinking per week was 0.97 (*SD* = 1.36) for men and 0.42 (*SD* = 0.78) for women.

There were several significant relationships between demographic variables and past-year drinker/abstainer status. Racial groups (white versus non-white) differed in past-year alcohol use, *X*^2^ (1, *N* = 558) = 10.44, *p* = 0.001. Among non-whites, 39% were past-year abstainers, compared to 24.6% of whites. There were also significant academic class differences, *X*^2^ (1, *N* = 557) = 51.59, *p*<0.001. Seniors were more likely to have been past-year drinkers (92.1%). In contrast, 56.2% of first-year students were past-year drinkers. Sophomores and juniors were also more likely than first-years to have consumed alcohol in the past year (78.7% and 81.6%, respectively). There were no significant sex differences in drinker/abstainer status.

### Interest in clinical trial

Only past-year drinkers were included in the primary analyses (*n* = 411). Distributions for continuous variables were examined first. Transformations were utilized when skewness values were greater than 3, as was the case for drinks per drinking day, binge drinking, negative consequences and peer drinking norms. Correlations were examined to determine whether any variables to be included in the logistic regression were strongly related. The only such relationship was drinks per drinking day with frequency of binge drinking, *r* = 0.62, *p*<0.001. While high, this correlation was under the 0.70 threshold thought to be indicative of concern regarding multicollinearity [44].

Interest in participation in a clinical trial of individual counseling plus naltrexone was indicated by 22.6% of past-year drinkers (*n* = 93). Level of interest was equivalent among past-year heavy drinkers (i.e., at least one heavy drinking day): 23.2% (85 out of 367 heavy drinkers). Quadratic terms for drinks per drinking day and frequency of binge drinking were both non-significant predictors and thus were removed from the model, leaving only linear terms for these two variables. A logistic regression testing the full model, including all variables identified a priori as potential predictors of interest in the clinical trial (i.e., the full model, Table 2) was found to provide a good fit to the data based on a significant chi-square goodness of fit test, *X*^2^ (15, *N* = 390) = 38.77, *p* = 0.001, and a non-significant Hosmer-Lemeshow [44] test, *X*^2^ (8, *N* = 390) = 5.73, *p* = 0.677. In the full model, two variables were significant (i.e., non-white race and smoking at least once per week) and another was a trend-level predictor of clinical trial interest (i.e., negative consequences) (Table 2). No model dimension had a condition index of 30 or greater, suggesting that multicollinearity was not a concern [45].

A second logistic regression including only variables found to predict interest in the clinical trial at a significant or trend level (*p* < 0.10, i.e., the reduced model) was also found to provide a good fit to the data based on a chi-square goodness of fit test, *X*^2^ (3, *N* = 408) = 30.59, *p*<0.001, and a non-significant Hosmer-Lemeshow test, *X*^2^ (6, *N* = 408) = 4.27, *p* = 0.640. In the reduced model, the negative consequences score was a significant predictor (Table 2).

Post-hoc chi-square analyses were conducted to test which negative consequences had significant relationships with interest in the clinical trial. Difficulty controlling alcohol consumption (i.e., impaired control), *X*^2^ (1, *N* = 408) = 7.18, *p* = 0.007; social consequences, *X*^2^ (1, *N* = 408) = 4.59, *p* = 0.032; and risky behaviors, *X*^2^ (1, *N* = 410) = 5.38, *p* = 0.016, were significantly related to interest in the clinical trial.

## Discussion

The present findings indicated greater interest in an alcohol reduction clinical trial involving pharmacotherapy and individual counseling among undergraduates who were non-white, current smokers and who reported more alcohol-related negative consequences. Clinical trial interest was not significantly related to alcohol consumption variables, perceived peer drinking norms, sex and past-year drug use.

Although non-white students reported greater interest in the clinical trial, they consumed less alcohol than whites, a finding that is consistent with prior studies. For example, in the Monitoring the Future study, African-American high school seniors reported less heavy episodic drinking than whites [26]. Young minority drinkers who compare their behaviors to same-race peers may see these behaviors as more problematic, motivating interest in drinking reduction. Findings suggest that stigma regarding substance use and abuse may be particularly strong among racial/ethnic minority groups [46], which may motivate some individuals to explore treatment options.

The results of the current study also suggest that being a cigarette smoker may motivate treatment interest. Given evidence for reciprocal effects between smoking and alcohol use via several mechanisms (e.g., cross-tolerance [47]), smokers may perceive their alcohol use to be more severe. Findings linking smoking to poorer alcohol treatment outcomes [32,48] suggest that drinking behavior change may be particularly difficult for smokers, thus increasing the likelihood that they will be open to formal treatment. Notably, there is evidence that naltrexone may be efficacious for alcohol use reduction in smokers [49–51].

The finding that a higher number of negative consequences predicted interest in the treatment study supports prior findings of positive relationships between alcohol problem severity and an inclination toward behavior change in young adults [6,8,10]. Other findings in this vein have been equivocal [7] and some studies have found negative relationships between alcohol problem severity and inclination toward behavior change in this population [12,13]. This pattern of findings suggests a complex relationship between alcohol problem severity and inclination toward drinking reduction that should be the subject of additional research. The relationship between interest in a clinical trial and self-reported impaired control over alcohol use was notable given prior findings that impaired control is a prospective predictor of alcohol-related problems in undergraduates [52,53]. Overall, findings from this and prior studies suggest at least some of the most problematic undergraduate drinkers may recognize a need to reduce their drinking.

Results of this survey are valuable because they provide information about undergraduates’ openness to pharmacotherapy for alcohol use reduction. Young adult heavy drinking is a considerable public health problem [2], motivation to change in this population tends to be limited [8], and current individual counseling approaches showsmall effect sizes and may be less efficacious for the heaviest drinkers [15]. Thus, there is a need for more intensive intervention options. Given that pharmacotherapy for mental health issues has become more common among undergraduates [5], medication represents a more intensive intervention option for alcohol reduction that may grow in popularity in coming years. Naltrexone is of particular interest given its demonstrated efficacy for drinking reduction in those not motivated to quit [18,54], an important consideration given that undergraduate drinkers tend not to be interested in stopping alcohol use outright [8]. However, given the low prescription rates for naltrexone [24], relatively few drinkers are likely to be aware of it. Thus, interest in enrollment in this clinical trial to test naltrexone plus individual counseling may provide an index of more general openneness to intensive drinking reduction intervention.

Due to the particularities of young adult heavy drinking and the challenges inherent in recruiting and retaining undergraduate drinkers in alcohol treatment and clinical trials, methods to engage this population are needed [9]. Surveys may be one useful approach in that they provide insight as to which characteristics may be associated with openness to behavior change. This information may be of use to professionals who work with undergraduate heavy drinkers. Advertisements for alcohol reduction efforts and the makeup of interventions themselves may be directed toward individuals with these characteristics.

## Limitations

Our desires to keep the survey brief precluded use of many established measures. Further, because monetary compensation is provided to participants in the clinical trial, our findings may not generalize to interest in programs offering little or no remuneration (e.g., student health services). It is worth noting though that the compensation was mentioned after the purpose and type of treatment were described. The wording also stated that compensation was provided “in the course of the study,” making clear that payment would not be in a lump sum. The rate of interest expressed by survey respondents was probably higher than for other interventions, but was still reported by less than ¼ of past-year drinkers. This suggests that, although the compensation probably contributed to students’ interest, it was not enticing enough to attract a majority of respondents. Thus, it seems unlikely that many students without some intrinsic motivation to change expressed interest in the trial due to the compensation alone.

The survey response rate was low compared to traditional pencil and paper surveys, though comparable to several recent, web-based substance-related surveys in undergraduates offering comparable compensation [39–43]. A web-based approach has the benefit of targeting a broader audience than traditional approaches. Further, even a non-representative subgroup from a random sample is likely to be more representative of the population from which it is drawn than a convenience sample (e.g., undergraduate psychology students). Experts have argued that “it is difficult to overestimate the importance of random sampling” [55].

Despite these limitations, this study provides important information bearing on the public health concern of heavy drinking in undergraduates by identifying predictors of interest in a promising treatment. We hope the present report leads to similar reports of predictors of interest in a variety of treatment options and research studies aimed at alcohol reduction in young adults.

## Conclusions

The present study had a number of other strengths including a relatively large number of respondents and a stratified, random sample. Further, this study was unique in that survey respondents who indicated interest in the trial were essentially engaging in an action step toward behavior change because this step initiated contact from the study staff. Most prior studies regarding treatment interest and inclination have concerned retrospective reports of treatment utilization [6,7,11] and/ or hypothetical future interest [7,8,10]. Further, little is known about undergraduates’ openness to medication for alcohol use reduction, thus findings from this survey help to address a gap in the literature.

## Figures and Tables

**Table 1:** Selected descriptives for the entire sample and for past year drinkers only.

Variable	Entire sample (*N* = 584)	Past-year drinkers only (*n* = 411)	Past-year heavy drinkers only (n = 367)

Percent male	39.8%	39.7%	40.9%

Percent Hispanic/Latin	11.7%	12.1%	13.8%

Percent other races/ethnicities	White: 75.7%	White: 79.1%	White: 79.2%
	Black: 7.3%	Black: 6.2%	Black: 4.7%
	Asian: 2.5%	Asian: 1.4%	Asian: 1.6%
	Amer. Indian: 0.7%	Amer. Indian: 0.7%	Amer. Indian: 0.5%
	multiple race: 4.4%	multiple race: 3.8%	multiple race: 3.8%
	other: 9.4%	other: 8.8%	other: 10.1%

Academic class breakdown	first: 41.2%	first: 32.4%	first: 31.3%
	sophomore: 25.3%	sophomore: 27.3%	sophomore: 27.8%
	junior: 20.1%	junior: 22.9%	junior: 22.6%
	senior/beyond: 13.4%	senior & beyond: 17.5%	senior & beyond: 18.3%

Pct. smoking cigarettes at least once/week	9.2%	12.2%	13.4%

Mean (SD) drinks per drinking day	3.16 (3.54)	4.34 (3.48)	7.64 (1.70)

Mean (SD) weekly binge drinking frequency	0.47 (0.98)	0.65 (1.10)	0.72 (1.14)

**Table 2:** Summary of Logistic Regression Analyses Predicting Interest in an Alcohol Reduction Clinical Trial of Naltrexone among University Undergraduates.

		Full Model				Reduced Model		

Variable	*B*	*SE B*	Odds ratio	95% CI	*B*	*SE B*	Odds ratio	95% CI

Drinks per drinking day	−0.45	0.67	0.64	0.17–2.37				
Frequency of binge drinking	0.96	0.65	0.38	0.11–1.38				
Negative consequences of alcohol	0.31#	0.18	1.36	0.96–1.94	0.35*	0.14	1.41	1.07–1.87
Mean of peer drinking norms items	0.01	0.16	1.01	0.74–1.40				
Smoking (smoker: 1, non-smoker: 0)	1.28***	0.37	3.60	1.79–6.62	1.24***	0.33	3.44	1.81–6.54
Past-year illegal drug use (yes: 1, no: 0)	−0.21	0.32	0.82	0.44–1.51				
Past-yr prescription abuse (yes:1, no:0)	0.60	0.46	1.83	0.74–4.54				
Current emotional/psychiatric condition	−1.16	0.91	0.31	0.05–1.87				
Race (white: 1, non-white: 0)	−0.85**	0.30	0.43	0.24–0.78	−0.93***	0.28	0.39	0.23–0.69
Sex (male: 1, female: 0)	−0.33	0.29	0.72	0.41–1.28				
Academic class: dummy coded								
sophomore	−0.70	0.35	0.50	0.25–0.98				
junior	−0.54	0.37	0.58	0.28–1.21				
senior	−0.39	0.42	0.68	0.30–1.56				
Living situation: dummy coded								
dormitory	−0.08	0.46	0.92	0.38–2.26				
residence with friends/roommates	0.11	0.54	1.12	0.39–3.20				
Constant	0.48				−1.09			

Only significant or trend level predictors of interest in the clinical trial (*p* ≤ 0.10) were included in the reduced model, with adjustments made for dummy coded variables. For “academic class,” freshman year was the reference group. For “living situation,” residence with parents was the reference group. “Smoker” was defined as reporting smoking at least once per week. Negative consequences were a sum score out of a possible 9.

# *p* ≤ 0.10,

* *p* ≤ 0.05,

** *p* ≤ 0.01,

*** *p* ≤ 0.001
